# Phase-sensitive T1 inversion recovery imaging and its impact on the detection of cortical demyelinating lesions in patients with multiple sclerosis

**DOI:** 10.1590/0100-3984.2023.56.4e3

**Published:** 2023

**Authors:** Fabiano Reis

**Affiliations:** 1 Associate Professor, Department of Anesthesiology, Oncology, and Radiology, Head of the Magnetic Resonance Sector at Hospital das Clínicas, Faculdade de Ciências Médicas da Universidade Estadual de Campinas (FCM-Unicamp), Campinas, SP, Brazil. Email: fabianoreis2@gmail.com.

Multiple sclerosis (MS) is the most common chronic inflammatory demyelinating disease of the central nervous system (CNS). It is one of the most relevant causes of neurological disability in young adults. Symptoms usually start between 20 and 40 years of age. There is a female preponderance, with a female:male ratio of 2-3:1^(1-4)^.

According to the 2017 McDonald criteria^(5)^, the diagnosis of MS depends on clinical or radiological evidence of CNS lesions that are disseminated in space and time. The dissemination in space criteria require one or more T2-weighted image (T2WI) hyperintense lesions that are characteristic of MS, measuring at least 3 mm on their longest axis, in two or more of the following regions^(5)^: periventricular; cortical or juxtacortical; infratentorial; and spinal cord. For dissemination in time, one of two conditions must be met^(5)^: a new T2WI-hyperintense or gadolinium-enhancing lesion in comparison with the baseline MRI scan; or the simultaneous presence of gadolinium-enhancing and non-enhancing lesions on any MRI scan.

Conventional MRI is not capable of demonstrating the true extent of pathological abnormalities occurring in MS. It can miss anomalies observed in non-conventional MRI and neuropathology studies^(6,7)^. Advances in neuroimaging, including new techniques used as adjuncts to conventional MRI, are significantly changing our capacity to diagnose and determine the prognosis of cases of MS.

Ultra-high-field (7.0-T) and very-high-field (3.0-T) MRI have been shown to perform better than high-field (1.5-T) MRI in demonstrating cortical gray matter involvement, which can occur in adults and children with MS^(8,9)^.

Phase-sensitive T1 inversion recovery imaging (PSIR) provides excellent contrast between white and gray matter, with better delineation at shorter scan times relative to conventional spin echo and T1-weighted fluid-attenuated inversion recovery (FLAIR) sequences^(10)^.

The article “Interrater reliability for the detection of cortical lesions on phase-sensitive inversion recovery magnetic resonance imaging in patients with multiple sclerosis”, authored by Caneda et al.^(11)^ and published in the current issue of Radiologia Brasileira, provides a valuable tool to assess PSIR sequences acquired to demonstrate cortical lesions in the relapsing-remitting MS phenotype, with an impact on the diagnosis and monitoring of patients with MS. Their results indicate that the performance of PSIR sequences is superior to that of conventional MRI sequences, particularly FLAIR sequences, for characterizing cortical lesions. The authors found good interrater agreement for the PSIR sequences, even between raters with markedly different levels of experience. To our knowledge, theirs is the first study to employ raters of different experience levels to assess interrater reliability for PSIR, thus avoiding training effects on the agreement coefficients. The authors also demonstrated that cortical lesions depicted by PSIR have an impact on the Expanded Disability Status Scale scores, corroborating the data previously reported by Nielsen et al.^(12)^.

If PSIR proves to be a clinically useful tool in the evaluation of patients with MS, there is a possibility that it will be included in the MRI protocols for MS. That could increase the efficacy of imaging in such patients.

## References

[1] Dobson R, Giovannoni G. (2019). Multiple sclerosis - a review. Eur J Neurol.

[2] Doshi A, Chataway J. (2016). Multiple sclerosis, a treatable disease. Clin Med (Lond).

[3] Oh J, Vidal-Jordana A, Montalban X. (2018). Multiple sclerosis: clinical aspects. Curr Opin Neurol.

[4] Tamanini JVG, Sabino JV, Cordeiro RA (2023). The role of MRI in differentiating demyelinating and inflammatory
(not infectious) myelopathies. Semin Ultrasound CT MR.

[5] Thompson AJ, Banwell BL, Barkhof F (2018). Diagnosis of multiple sclerosis: 2017 revisions of the McDonald
criteria. Lancet Neurol.

[6] Kolappan M, Henderson APD, Jenkins TM (2009). Assessing structure and function of the afferent visual pathway
in multiple sclerosis and associated optic neuritis. J Neurol.

[7] Castro SMC, Damasceno A, Damasceno BP (2013). Visual pathway abnormalities were found in most multiple
sclerosis patients despite history of previous optic
neuritis. Arq Neuropsiquiatr.

[8] Brenton JN, Kammeyer R, Gluck L (2020). Multiple sclerosis in children: current and emerging
concepts. Semin Neurol.

[9] Pereira FV, Jarry VM, Castro JTS (2021). Pediatric inflammatory demyelinating disorders and mimickers: how
to differentiate with MRI?. Autoimmun Rev.

[10] Hou P, Hasan KM, Sitton CW (2005). Phase-sensitive T1 inversion recovery imaging: a time-efficient
interleaved technique for improved tissue contrast in
neuroimaging. AJNR Am J Neuroradiol.

[11] Caneda MAG, Rizzo MRL, Furlin G (2023). Interrater reliability for the detection of cortical lesions on
phase-sensitive inversion recovery magnetic resonance imaging in patients
with multiple sclerosis. Radiol Bras.

[12] Nielsen AS, Kinkel RP, Madigan N (2013). Contribution of cortical lesion subtypes at 7T MRI to physical
and cognitive performance in MS. Neurology.

